# Research Review: What we have learned about the endocannabinoid system in developmental psychopathology

**DOI:** 10.1111/jcpp.70006

**Published:** 2025-07-10

**Authors:** Ryann C. Tansey, Marc D. Ferger, Hilary A. Marusak, Leah M. Mayo

**Affiliations:** ^1^ Department of Psychiatry Mathison Centre for Mental Health Research and Education, and Hotchkiss Brain Institute, University of Calgary Calgary AB Canada; ^2^ Department of Child and Adolescent Psychiatry, Psychosomatics and Psychotherapy Faculty of Medicine and University Hospital Cologne, University of Cologne Cologne Germany; ^3^ Institute of Experimental and Clinical Pharmacology, Toxicology and Pharmacology of Natural Products Ulm University Medical Center Ulm Germany; ^4^ Department for Child and Adolescent Psychiatry/Psychotherapy University of Ulm Ulm Germany; ^5^ Department of Psychiatry and Behavioral Neurosciences Wayne State University School of Medicine Detroit MI USA

**Keywords:** Endocannabinoid, 2‐arachidonoylglycerol, anandamide, psychopathology, development

## Abstract

**Background:**

The endocannabinoid (eCB) system, the primary target of cannabis, has gained significant attention as a potential novel therapeutic approach for treating a range of psychiatric disorders characterized by dysregulation of stress, emotion, and social behavior. The use of cannabis itself as a pharmacotherapeutic in children and adolescents is limited due to various constraints, including legal status, stigma, and real or perceived negative side effects. Thus, compounds that target the eCB system without the notable unwanted effects of cannabis may offer a more viable approach for developing populations.

**Methods:**

In this narrative review, we provide an overview of the eCB system, summarizing its function throughout development and its potential contribution to psychopathology in children and adolescents. We highlight evidence of its behavioral role and the dysregulation of this system in various psychiatric disorders. Finally, we summarize current investigations into pharmacological and nonpharmacological therapeutic interventions designed to target the eCB system.

**Conclusions:**

The eCB system may offer an innovative target for treatments of various psychiatric disorders in child and adolescent populations. However, more research is needed to understand the nuanced developmental trajectory of this system and to determine whether existing compounds are safe and effective for use in these populations.

## Introduction

Cannabis and cannabis‐based therapeutics, including the main cannabinoids Δ9‐tetrahydrocannabinol (THC) and cannabidiol (CBD), have long been of interest in psychiatry. While there have been promising advances in cannabinoid‐based treatments for adults, their use in children and adolescents is limited, in part due to the reported negative impacts on neurodevelopment (Albaugh et al., 2021). This underscores the pressing need to explore alternative interventions that safely mimic the therapeutic effects of cannabis‐based treatments in younger populations.

Cannabinoids interact with the endocannabinoid (eCB) system, which regulates a wide range of homeostatic processes, including stress, emotion, pain, sleep, and feeding (Ligresti, De Petrocellis, & Di Marzo, 2016). THC, which is responsible for the intoxicating effects of the drug (Ligresti et al., 2016), is a high‐affinity partial agonist at the cannabinoid receptors CB1 and CB2, which were first isolated in the late 1980s (Devane, Dysarz, Johnson, Melvin, & Howlett, 1988; Munro, Thomas, & Abu‐Shaar, 1993). The discovery of cannabinoid receptors encouraged the search for their endogenous ligands. Subsequently, N‐arachidonoylethanolamine, more commonly referred to as anandamide (AEA), and 2‐arachidonoylglycerol (2‐AG) were discovered in the early 1990s (Devane et al., 1992; Mechoulam et al., 1995). AEA is a partial agonist at CB1 receptors, while 2‐AG is a full agonist. 2‐AG is more abundant in both the central nervous system and peripheral organs than AEA, with concentrations ranging from 100 to 1,000 times higher (Gonsiorek et al., 2000). The elucidation of this system opened avenues for the development of pharmaceuticals that could provide the benefits of cannabis without intoxication.

In this review, we provide a comprehensive overview of the eCB system, its role and intersection with psychopathology during development, and current evidence for its involvement in behavior in individuals with and without psychiatric disorders. While several clinically relevant psychopathologies in childhood and adolescence have been linked to alterations in the eCB system, other prevalent psychiatric disorders in pediatric populations have not demonstrated such associations and are therefore excluded from this review. Given that the majority of psychiatric disorders have an age of onset during the first two decades of life (McGrath et al., 2023), research investigating the role of the eCB system during this period could lead to treatments tailored to the developing brain and prevention strategies to stem the etiology of these disorders.

## Overview of the eCB system

**Figure 1:**
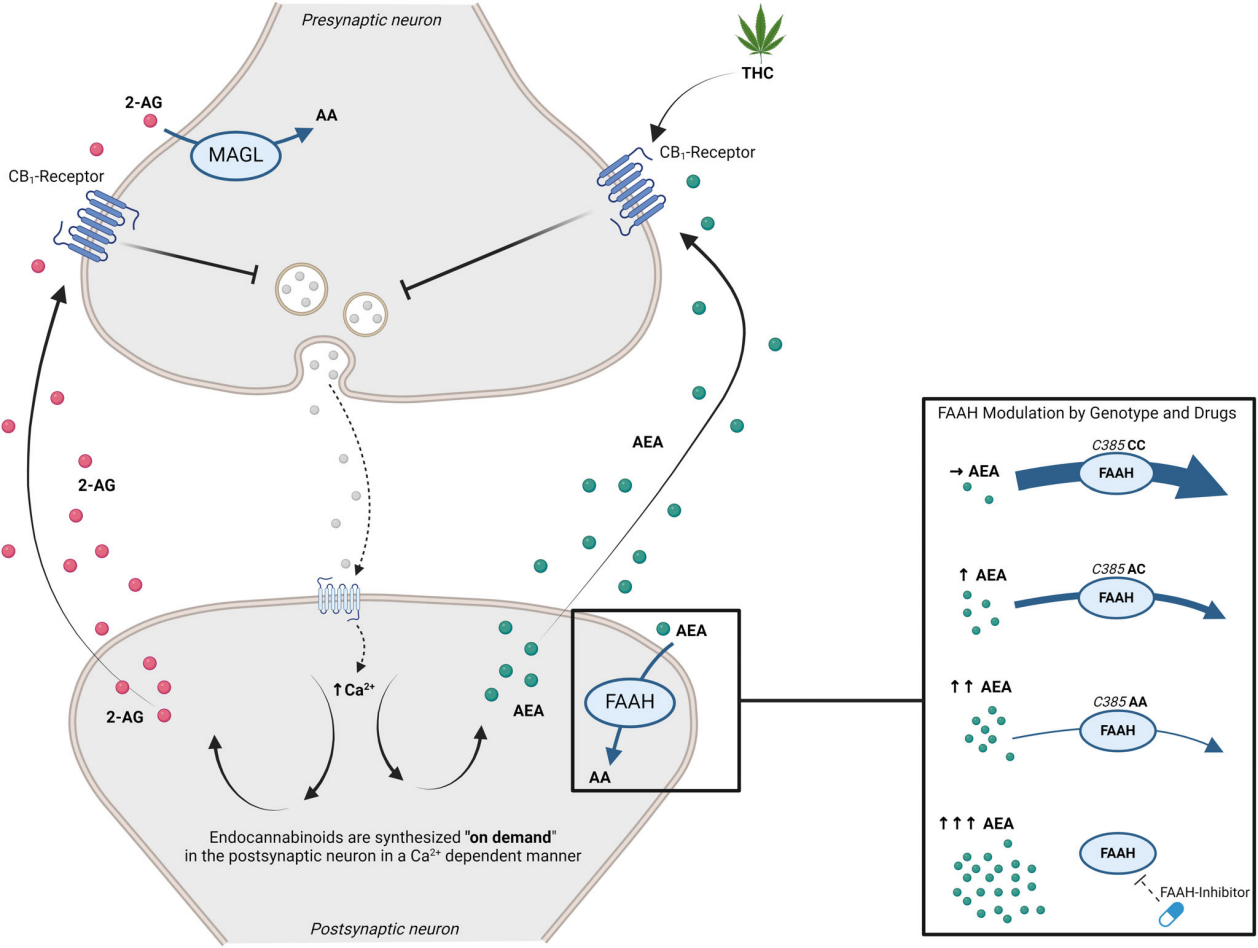
Graphical representation of eCB signaling at the synapse. Stimulation of the postsynaptic neuron by the pre‐synaptic neuron leads to the synthesis of the eCB ligands anandamide (AEA) and 2‐arachidonoylglycerol (2‐AG). These travel across the synapse in a retrograde fashion to bind to cannabinoid receptor 1 (CB1) on the pre‐synaptic neuron, inhibiting its activity. The box on the bottom right illustrates the effects of the FAAH C385A SNP and FAAH inhibitors on FAAH activity. The presence of the A allele is associated with reduced FAAH activity, resulting in higher peripheral AEA concentrations, particularly in individuals homozygous for the A allele. The highest peripheral AEA concentrations are observed when FAAH activity is pharmacologically inhibited. Figure created with Biorender.

The eCB system has several features that differentiate it from classic neurotransmitter systems, as summarized in Figure 1. eCB signaling operates in a homeostatic retrograde fashion (Castillo, Younts, Chávez, & Hashimotodani, 2012). In response to repeated activation, the postsynaptic neuron releases eCB ligands, which travel across the synaptic cleft, bind to CB1 receptors on the pre‐synaptic cell, and trigger an overall inhibition of synaptic activity. The net synaptic effect of eCB signaling is dependent on the pre‐synaptic neuron type (Scheyer, Yasmin, Naskar, & Patel, 2023): for excitatory neurons, CB1 activation results in an overall decrease of transmission (Kreitzer & Regehr, 2001); while in inhibitory neurons, it results in an overall increase of transmission (Wilson & Nicoll, 2001). Unlike other neurotransmitters, which are typically stored in vesicles for future release, the eCBs are synthesized ‘on demand’ (Blankman & Cravatt, 2013). The enzymes fatty acid amide hydrolase (FAAH) and monoacylglycerol lipase (MAGL) are responsible for the degradation of AEA and 2‐AG, respectively (Blankman & Cravatt, 2013; Deutsch & Chin, 1993). Navigating the intricate pathways of eCB metabolism presents challenges due to the complex synthesis and degradation processes of AEA and 2‐AG.

While interventions targeting the biosynthesis pathways of AEA and 2‐AG remain elusive, the identification of compounds that inhibit FAAH and MAGL offers promising prospects. FAAH and MAGL inhibitors can increase brain levels of AEA and 2‐AG, respectively (Ahn et al., 2011; Long et al., 2009), thus amplifying the physiologically relevant, endogenous eCB signal at specific neuronal targets. This contrasts with the administration of exogenous CB1 agonists, such as THC, which bind to CB1 receptors across the brain indiscriminately. As such, facilitating eCB function through enzyme manipulation may provide therapeutic opportunities that are not otherwise feasible with exogenous CB1 agonist administration.

Importantly, dysregulated eCB function has been implicated in multiple psychiatric disorders (Gowatch et al., 2024), including posttraumatic stress disorder (PTSD; Mayo, Rabinak, Hill, & Heilig, 2022), schizophrenia (Zamberletti & Rubino, 2021), anxiety (Lutz, Marsicano, Maldonado, & Hillard, 2015; Petrie, Nastase, Aukema, & Hill, 2021), depression (Gorzalka & Hill, 2011), and autism (De Pol & Kolla, 2021). Consequently, pharmacological alteration of eCB signaling may present a novel therapeutic option for a variety of psychiatric concerns.

Evidence for the role of eCBs in human behavior has been drawn from studies measuring natural variation in their levels, as well as pharmacological studies that target eCB signaling. Genetic variants in the eCB system appear to play a role in a number of neurological and psychiatric symptoms (Smith, Stanley, Foss, Boles, & McKernan, 2017). One source of variation in AEA levels is a common loss‐of‐function single‐nucleotide polymorphism (SNP; rs324420 *FAAH* 385C→A), of which ~37% of the population carry at least one allele copy, and ~5% two copies, though this varies based on race (Sherry, Ward, & Sirotkin, 1999). This mutation results in reduced enzymatic activity and lower cellular expression levels (Chiang, Gerber, Sipe, & Cravatt, 2004), and consequently higher AEA (Mayo et al., 2020). AA homozygote individuals have the highest measurable levels of AEA, whereas AC heterozygotes have levels greater than wild‐type but less than AA homozygotes (see Figure 1). This variation offers a valuable means to study the eCB system in developing populations without requiring pharmacological interventions or invasive blood sampling.

A complementary yet distinct approach in adults involves pharmacological manipulation to acutely raise eCB levels. AEA levels can be increased through the administration of a FAAH inhibitor, which results in a 10‐fold increase in peripheral AEA levels, compared with the ~ 30% increase seen with the *FAAH* 385C→A (rs324420) mutation (Mayo et al., 2020; Mayo, Asratian, Lindé, Holm, et al., 2020). Similarly, 2‐AG levels can be pharmacologically increased through the administration of a MAGL inhibitor, although clinical research into the effects of this class of compounds is more limited. These methodologies have enabled the study of the effects of eCBs on stress, fear, and social behavior in adult human subjects.

Much of the literature investigating eCBs and behavioral phenotypes is mixed. It is important to note that eCB function is very sensitive to perturbations by a number of *in* and ex vivo factors, including food intake, time of day, and room temperature (Hillard, 2018). Differences in these methodological considerations could serve as an important source of variation between findings and highlight the importance of conducting more rigorously controlled research in human populations.

## Development of the eCB system

Childhood and adolescence are key periods of neurodevelopmental susceptibility wherein alterations can have lasting impacts on outcomes later in life (Otto et al., 2021). Adolescents are particularly vulnerable to the negative effects of stress, which can have repercussions for future mental health (Andersen & Teicher, 2008). During adolescence, important structural characteristics of the brain are in flux, including the functional connections between areas such as the amygdala, prefrontal cortex, hippocampus, and nucleus accumbens (Andersen & Teicher, 2008). The rapid rate of change during this period makes adolescents particularly sensitive to perturbations from environmental exposures such as early life adversity, which is very common (Lopez et al., 2021). This contributes to an elevated risk of various mental health issues throughout the lifespan (Fujiwara, 2022). In this section, we outline what is currently known about the development of the eCB system and its contributions to mental health in youth.

The developmental trajectories of the eCB system appear to follow complex and fluctuating patterns. The interplay between eCB signaling and stress is bidirectional: while eCBs play a role in regulating the stress response, the eCB system itself can be adversely affected by stressful experiences (Morena, Patel, Bains, & Hill, 2016). While animal models have provided detailed insights into eCB circuitry maturation, there is a paucity of literature translating these findings into humans (for a detailed overview of eCB fluctuations across development in preclinical models, see Meyer, Lee, & Gee, 2018).

The eCB system appears to play a functional role in critical developmental periods. Current evidence suggests that CB1 receptor expression is observable in the human brain as early as 14 weeks gestation (Mato, Del Olmo, & Pazos, 2003; Wang, Dow‐Edwards, Keller, & Hurd, 2003). However, rodent studies have detected CB1 expression as early as gestational day 11, equivalent to weeks 5–6 in human embryos (Buckley, Hansson, Harta, & Mezey, 1997; Wu, Jew, & Lu, 2011), suggesting that CB1 receptor expression may emerge even earlier than originally thought. The expression of the *CNR1* gene, which encodes the CB1 receptor, peaks in the human fetal prefrontal cortex during the second trimester and declines after birth (Tao et al., 2020). Further, CB1 receptors have been shown to activate signal transduction cascades from early developmental stages (gestational days 14–21 in rat) and are transiently expressed in structures where they are absent in the adult brain, suggesting a functional role in fetal brain development (Fernández‐Ruiz, Berrendero, Hernández, & Ramos, 2000).

CB1 receptor expression also fluctuates during the first two decades of postnatal life. In postnatal animal models, peak CB1 receptor expression in the prefrontal cortex and limbic areas occurs in early adolescence (Heng, Beverley, Steiner, & Tseng, 2011; Meyer et al., 2018), followed by a decrease into adulthood. However, there may not be a straightforward translation between the developmental trajectories observed in animals and humans. A recent study highlighted the number of cross‐species differences in eCB expression in the periphery across mice, rats, and nonhuman primates (Rosado‐Franco et al., 2024), emphasizing the potential pitfalls of overgeneralizing preclinical findings into humans. Postmortem studies of human tissue have found that CB1 receptor expression in the prefrontal cortex peaks much earlier in life, around infancy and toddlerhood (Long, Lind, Webster, & Weickert, 2012). Similarly, CB1 receptor expression was higher in infancy compared with adulthood in the human midbrain, an area implicated in pain processing (Kwok et al., 2017). This preliminary evidence suggests that while both animal and human research points to the eCB system being a key player in brain development, the developmental trajectories observed in model species may not uniformly apply to humans, emphasizing the importance of further research in humans.

Given the potential differences in eCB development between species, it is important to identify where findings from disparate research modalities converge. A consistent theme emerging from both animal and human studies is that AEA and 2‐AG exhibit divergent developmental dynamics. In the human dorsolateral prefrontal cortex (dlPFC), both FAAH, which degrades AEA, and N‐acylphosphatidylethanolamine‐specific phospholipase D (NAPE‐PLD), which synthesizes AEA, increase in expression from infancy to adulthood, potentially peaking in early adulthood (Long et al., 2012). In contrast, the enzymes regulating 2‐AG expression show more complex trajectories. MAGL, the primary 2‐AG degradation enzyme, peaks in the dlPFC during infancy and then declines into adulthood. Conversely, diacylglycerol lipase (DAGL), one of the 2‐AG synthesis enzymes, does not peak until adolescence (Long et al., 2012). This suggests that 2‐AG levels in the human prefrontal cortex may reach their highest point during adolescence. Further, recent evidence from animal models indicates that biological sex may influence the trajectories of AEA and 2‐AG signaling (Bernabeu et al., 2023; Lee, Hill, & Lee, 2016). Therefore, it is important to characterize the developmental trajectories of AEA and 2‐AG in youth, considering biological factors such as sex.

### Links between eCB development and psychopathology

Several key studies have demonstrated an important relationship between eCB system development and behavioral phenotypes linked to psychiatric risk. Using data from the Adolescent Brain Cognitive Development Study® (Casey et al., 2018; Jernigan, Brown, & Dowling, 2018), we previously investigated the associations between the *FAAH* C385A polymorphism, anxiety symptoms, and frontolimbic white matter pathways (structural connections) in children aged 9–11 years (Marusak, Evanski, Desai, & Rabinak, 2022). Fractional anisotropy (FA), a common metric reflecting various microstructural characteristics of white matter, was used to examine white matter integrity (Kochunov et al., 2012). Compared with C‐allele homozygotes, children with the *FAAH* 385A allele – associated with higher AEA levels – exhibited lower FA of the left fornix and parahippocampal cingulum, which are frontolimbic pathways critically involved in stress and anxiety regulation (Bubb, Metzler‐Baddeley, & Aggleton, 2018; Senova, Fomenko, Gondard, & Lozano, 2020). Notably, *FAAH* did not directly predict anxiety symptoms in this preadolescent sample. Instead, left fornix FA mediated the genotype–anxiety relationship, with higher FA associated with greater anxiety. These findings align with prior research suggesting that direct associations between *FAAH* and anxiety symptoms emerge later, during adolescence. For instance, in adolescents (12+ years), *FAAH C385A* was linked to lower anxiety and increased FA in the uncinate fasciculus (Gee et al., 2016) – a frontolimbic pathway implicated in memory, cognitive control, and reward processing (Olson, Heide, Alm, & Vyas, 2015). The *FAAH C385A* variant may influence frontolimbic white matter integrity in youth, shaping psychiatric risk over development. However, the direction of associations between *FAAH*, FA, and anxiety symptoms may vary by both tract and developmental stage.

More recent reports using the ABCD Study® data provide further insights into the relationship between the *FAAH C385A* polymorphism, brain function, and mental health. We again found no significant associations between FAAH genotype and anxiety symptoms, or *FAAH C385A* and functional activation of the nucleus accumbens or amygdala (key regions associated with reward and emotion‐related processing, respectively) during an emotional *n*‐back task (Desai et al., 2024). However, children and adolescents homozygous for the A allele had significantly lower depressive symptoms than the CC homozygotes and AC heterozygotes. In another study, ABCD Study® data were used to identify a large, spatially distributed network that differed based on FAAH genotype (Sisk et al., 2022). CC homozygotes whose functional connectome fingerprints more greatly resembled A allele carriers across this distributed network had lower anxiety, suggesting that the functional connectivity profile of A allele carriers may represent a resilience factor against anxiety. Together, these findings support the idea that genetic variation in *FAAH* during development has widespread, complex impacts across both structure and function of the brain, which may be relevant to mental health.

## Behavioral and psychiatric correlates of eCB function

### Behavioral studies in nonclinical controls

Dysregulation of fear‐ and stress‐related mechanisms is thought to underlie numerous psychiatric conditions (Shin & Liberzon, 2010). Preclinical evidence for the role of eCBs in regulating stress and fear behaviors has been replicated in human studies; however, behavioral studies detailing the effects of eCBs on stress and fear in child and adolescent populations are more scarce. In nonclinical adults, individuals homozygous for the *FAAH* 385A allele demonstrate enhanced fear extinction and protection against stress‐induced negative affect (Mayo, Asratian, Lindé, Holm, et al., 2020; Ney et al., 2023; Spohrs et al., 2022), as well as lower levels of anxiety than CC homozygotes (Dincheva et al., 2015; Spohrs, Ulrich, Grön, Plener, & Abler, 2022). Other studies have examined the effects of *FAAH C385A* on amygdala and ventromedial prefrontal cortex (vmPFC) activation – regions thought to play an important role in the regulation of fear‐related learning and memory, particularly fear extinction (Bukalo et al., 2015). In studies of nonclinical adult samples, *FAAH* 385A carriers show lower activation of the amygdala in response to threat cues (Hariri et al., 2009) and during early extinction recall (Zabik et al., 2022), as well as enhanced functional connectivity between the amygdala and vmPFC at rest (Dincheva et al., 2015). Research using both fMRI and a positron emission tomography (PET) radiotracer for FAAH found that lower FAAH levels in the amygdala were associated with lower amygdala response to a threatening social cue, as well as higher resting‐state functional connectivity between the amygdala and vmPFC (Green et al., 2021, 2022). Together, these findings suggest that FAAH may regulate frontolimbic brain circuits involved in fear and emotion‐related processing.

These observational neuroimaging studies appear to be replicated in pharmacological studies. We previously demonstrated that pharmacological FAAH inhibition resulted in greater recall of fear extinction memory, attenuation of subjective and autonomic stress reactivity, and protection against stress‐induced negative affect (Mayo, Asratian, Lindé, Morena, et al., 2020). Neuroimaging data also showed that FAAH inhibition reduced activation in the anterior cingulate cortex, insula, and amygdala in response to emotional faces (Paulus et al., 2021). Enhancing AEA signaling through FAAH inhibition may help protect against the negative effects of stress‐ and fear‐related behaviors.

The eCB system may also confer resilience to the negative consequences of early life adversity. We previously demonstrated that childhood maltreatment is associated with an increased risk for developing a substance use disorder (SUD) in adulthood (Capusan et al., 2021), which fits with the broader literature. However, in another study, we found that adults with documented childhood trauma but *no* history of SUD had higher plasma AEA levels compared with nonclinical controls. These elevated AEA levels were associated with greater vmPFC activity during emotional conflict monitoring (Perini et al., 2023), suggesting that circulating AEA concentrations may be linked to resilience, potentially through enhanced emotion regulation abilities. However, further experimental research is needed to confirm this hypothesis and to better characterize the developmental trajectory of these effects, particularly in adolescence.

In addition, 2‐AG has also been implicated in stress and fear processing. Preliminary evidence suggests that 2‐AG may play an important role in the attenuation of fear generalization, a phenomenon involving the transference of fear responses to neutral stimuli that is commonly observed in trauma‐related disorders (Bedse, Hill, & Patel, 2020). Following stress induction, changes in peripheral 2‐AG concentrations, measured in both blood and saliva, have been observed in adults (Ney, Stone, et al., 2021; Spohrs, Prost, et al., 2022), but a recent meta‐analysis did not find consistent evidence supporting this trend (Gowatch et al., 2024). To determine the therapeutic potential of eCBs in youth, it will be imperative to conduct well‐controlled, age‐appropriate behavioral studies on stress and fear processing.

### Studies in psychiatric disorders

#### PTSD

While there is evidence that elevated AEA (occurring naturally or pharmacologically) is associated with protection against stress‐ and fear‐effects in nonclinical populations (Mayo, Asratian, Lindé, Holm, et al., 2020; Mayo, Asratian, Lindé, Morena, et al., 2020), the relationship in PTSD and trauma‐exposed individuals is more complex. In a study investigating fear conditioning in PTSD individuals, the *FAAH C385A* allele was associated with higher initial skin conductance responses to the conditioned stimulus (CS+), as well as increased physiological arousal to the conditioned stimulus that had not been paired with the aversive stimulus (CS‐), which suggests that there may have been fear generalization (Ney, Matthews, et al., 2021). Another study measured blood eCB levels at hospital admission immediately following traumatic injury and followed up with adult participants 6–8 months later. The authors found that higher AEA concentrations immediately following the traumatic event were associated with a greater likelihood of PTSD diagnosis at follow‐up, and individuals homozygous for the *FAAH* A allele had significantly higher PTSD symptoms at follow‐up compared with CC and AC genotypes (deRoon‐Cassini et al., 2022). Taken together, this suggests that FAAH and AEA may play different roles in stress and fear responses in those with PTSD compared with controls.

Race and ethnicity may also play a role in the interaction between AEA and trauma. Our recent study of urban youth (~60% racial minority) with high levels of trauma exposure (90% of participants) found that higher circulating AEA concentrations were associated with more severe PTSD symptoms, particularly hyperarousal (Marusak et al., 2024). Additionally, youth carrying the *FAAH* A allele exhibited higher AEA concentrations and greater PTSD severity. This highlights the need for further research in trauma‐exposed youth and diverse populations to better understand these complex interactions, particularly given the high prevalence of trauma exposure among racially minoritized youth (Pumariega, Jo, Beck, & Rahmani, 2022).

#### Social anxiety disorder (SAD)

eCBs have been implicated in social processing (Karhson, Hardan, & Parker, 2016). There has been growing interest in whether the eCB system could serve as a useful therapeutic target for SAD. A PET imaging study found that adults with SAD had elevated levels of whole brain FAAH when compared with controls (Ahmed et al., 2020), which may indicate lower AEA concentrations. Clinical trials have explored whether FAAH inhibitors are effective in adults with SAD, though suboptimal dosing potentially contributed to only marginal improvements (Schmidt et al., 2021). Nonetheless, there may be a preliminary signal for the use of eCB‐based therapies in SAD which should be explored more thoroughly.

#### Autism spectrum disorder (ASD)

Unlike other psychiatric disorders, preliminary research into the eCB system in ASD has included studies in child and adolescent participants. A recent scoping review found alterations in peripheral eCB levels in children with ASD across several studies (De Pol & Kolla, 2021). Peripheral AEA levels were consistently lower in ASD individuals compared with controls, while the findings for 2‐AG were mixed. This suggests that there may be eCB system alterations involved in ASD, though more research is required.

#### Depression

The eCB system has been investigated as a potential target for major depressive disorder (MDD) (Gorzalka & Hill, 2011). One major catalyst of this area of research was the observation that the weight‐loss drug rimonabant, which blocks CB1 receptor action and downregulates eCB signaling, had the unintended effect of increasing depression and anxiety symptoms in a clinical trial for obesity (Nissen et al., 2008). Since then, multiple studies have demonstrated that the eCB system is altered in individuals with MDD compared with controls, though the patterns of alteration have been variable (McWhirter, Bugarcic, Steel, & Schloss, 2024). Early reports found that serum AEA and 2‐AG were lower in individuals with depression (Hill, Miller, Carrier, Gorzalka, & Hillard, 2009; Hill, Miller, Ho, Gorzalka, & Hillard, 2008). However, more recent studies found that both AEA and 2‐AG blood levels were higher in individuals with depression (Mazurka et al., 2024; Obermanns et al., 2023), and another found no significant differences in the serum concentrations of AEA or 2‐AG in patients with minor or MDD compared with controls (Ho, Hill, Miller, Gorzalka, & Hillard, 2012).

Other alterations in eCB signaling pathways have also been implicated in depression. In one study, there was an increase of CB1 receptor density in the dlPFC of individuals with depression who died by suicide (Hungund et al., 2004). Another study found reduced CB1 receptor‐positive glial cells in postmortem anterior cingulate cortex tissue of individuals with MDD (Koethe et al., 2007). Thirty‐seven genetic mutations in the eCB signaling pathway were found to be more frequent in individuals with MDD compared with controls, though none were related to FAAH, MAGL, or CNR1 (Xu et al., 2023). More research is needed to fully understand the complex relationships between eCB and the emergence of MDD.

#### Borderline personality disorder (BPD) and nonsuicidal self‐injury (NSSI)

Research into the role of the eCB system in BPD has been mixed. In a pilot study, patients with BPD had reduced AEA concentrations in hair compared with controls (Wingenfeld et al., 2018), and a PET imaging study has shown elevated FAAH levels in the PFC of BPD patients (Kolla et al., 2020), suggesting lower concentrations of AEA in this region. In contrast, others have reported higher serum AEA concentrations in patients with BPD (Schaefer et al., 2014). A more recent study observed elevated plasma AEA levels in patients diagnosed with BPD, but notably, the *FAAH* genotype appears to play a crucial role in this finding (Spohrs et al., 2023). Unlike the control group, there was no significant difference in AEA levels for AA homozygotes compared with CC homozygotes in the BPD group, and the difference between groups was mainly driven by higher levels of AEA in the BPD CC group compared with the control CC group, suggesting that the relationship between AEA function and FAAH genotype may be altered in the disorder.

While not exclusive to BPD, recurrent nonsuicidal self‐injury (NSSI) is one of the clinical symptoms of BPD that can contribute to a diagnosis (American Psychiatric Association, 2013). We recently demonstrated that female adolescents in a specialized outpatient clinic exhibiting nonsuicidal self‐injurious behavior (NSSI) had lower AEA levels compared with age‐matched peers not exhibiting NSSI, regardless of childhood maltreatment history (Ferger, Sigrist, Brodesser, Kaess, & Koenig, 2024). Additionally, lower AEA levels were associated with a greater frequency of NSSI and a greater severity of childhood maltreatment. This research suggests that eCB processes, especially those related to AEA, may play a role in BPD and NSSI behaviors.

#### Obsessive‐compulsive disorder (OCD)

Given that exposure therapy – based on principles of fear extinction – is one of the established treatments for OCD (Berg, Webler, Klein, & Kushner, 2024), exploring the impact of eCB signaling on extinction processes could be particularly relevant. Preclinical research supports the idea that increasing eCB signaling can enhance extinction learning (Gunduz‐Cinar et al., 2023), which could potentially translate into human treatments for OCD. Human research investigating the eCB system in OCD remains scarce. However, a recent study found that levels of AEA and 2‐AG synthesizing enzymes were lower in adults with OCD compared with controls (Bellia et al., 2024). This suggests that alterations in eCB synthesis may be associated with OCD pathology. Given these findings, further research into the eCB system's role in OCD, particularly in children and adolescents, is warranted.

#### Schizophrenia

The relationship between adolescent cannabis use and the onset of psychosis in individuals with a predisposition to schizophrenia is well‐documented (Malone, Hill, & Rubino, 2010), and implicates the eCB system in the disorder. Although schizophrenia often manifests in early adulthood, structural and functional neurodevelopmental alterations relevant to the disorder can be observed as early as the adolescent and even prenatal periods in individuals later diagnosed with schizophrenia (Zamberletti & Rubino, 2021). A meta‐analysis found higher concentrations of AEA in the cerebrospinal fluid and in the blood of people with schizophrenia compared with healthy controls (Minichino et al., 2019). Research on the role of eCB signaling and neurodevelopment in schizophrenia has predominantly been conducted in animal models. For instance, animal studies have demonstrated that disruptions in eCB signaling can impact neurodevelopmental processes associated with schizophrenia, such as synaptic plasticity and neural connectivity (Zamberletti & Rubino, 2021). However, there is a notable scarcity of research focusing on the interactions between eCBs and schizophrenia during human development.

## Clinical interventions targeting the eCB system in psychiatry

### Pharmacological interventions

The development of FAAH inhibitors was stalled for a brief period of time following serious adverse events, including the death of one participant, during a Phase I multiple‐rising dose clinical trial involving the drug BIA‐10‐2,474 (Kerbrat et al., 2016). Subsequent investigations found that the adverse events were due to off‐target effects of that specific molecule, and not attributable to a class effect of FAAH inhibitors (van Esbroeck et al., 2017). FAAH inhibitor trials slowly recommenced; however, many trials that were paused during these investigations were ultimately left uncompleted.

In general, results from clinical trials in psychiatric conditions are mixed. Trials in PTSD (NCT05178316 and Eudra‐CT 2020‐001965‐36) and depression (NCT02498392 and EudraCT 2008‐001718‐26) failed to meet their primary endpoints. For cannabis use disorder, one of two trials showed that the FAAH inhibitor was able to reduce cannabis use 4 weeks after the commencement of the study (D'Souza et al., 2019). In a large (*n* = 228), multisite RCT assessing FAAH inhibitor efficacy in cannabis use disorder (NCT03386487) that concluded in 2023, 8 weeks of therapy with a FAAH inhibitor did not show a reduction in self‐reported cannabis use compared with placebo, though the results have not yet been published. As previously mentioned, one clinical trial investigating FAAH inhibition in SAD did not meet its primary endpoint, but exploratory analyses showed that there may be a dose‐dependent effect of FAAH inhibition in the disorder that warrants further investigation (Schmidt et al., 2021; NCT02432703). While almost all psychiatric FAAH inhibitor trials have been conducted in adults, there is one study that included adolescents that investigated the drug's efficacy in ASD. While it failed to meet the primary end point, the treatment group showed better responses to secondary end points such as domain‐specific core symptoms, repetitive behaviors, and anxiety (Klein et al., 2025).

Tourette syndrome has also received notable attention as a target for eCB‐based therapies. While a study with a FAAH inhibitor was terminated because no funding was available (NCT02134080), the results of an initial Phase 1b clinical trial with a MAGL inhibitor in adult patients demonstrated a significant reduction in premonitory urges, along with a significant effect on tic severity (Müller‐ Vahl et al., 2022). These encouraging findings prompted the initiation of a phase 2 clinical trial. However, the trial failed to achieve its primary end point of a significant change in tic severity compared with placebo (Müller‐ Vahl et al., 2021).

### Nonpharmacological interventions

Given the potential limitations and risks associated with pharmacological interventions during sensitive developmental periods, nonpharmacological strategies that modulate the eCB system may represent a promising area of research. One such approach is acute exercise, which reliably elevates eCB levels in both humans and nonhuman animals (Desai et al., 2022). Our recent systematic review and meta‐analysis revealed that acute exercise consistently increases both AEA and 2‐AG levels across various modalities (e.g., cycling, swimming) and species, including in individuals with physical and mental disorders. Environmental conditions may amplify these effects, as a strenuous hike at higher altitudes is associated with greater elevations in blood AEA concentrations compared with exercise at lower altitudes (Feuerecker et al., 2012). This suggests that hypoxic stress may amplify exercise‐related increases in AEA, although the isolated effects of hypoxia on eCB signaling remain unclear.

Exercise can also facilitate other eCB‐mediated processes. Aerobic exercise, when administered shortly after fear extinction, can enhance the consolidation of fear extinction learning (Crombie, Sartin‐Tarm, Sellnow, Ahrenholtz, Lee, Matalamaki, Adams, et al., 2021a), which may be mediated through elevations in AEA levels (Crombie, Sartin‐Tarm, Sellnow, Ahrenholtz, Lee, Matalamaki, Almassi, et al., 2021b). Further studies have also observed acute elevations in AEA following singing (Stone et al., 2018) highlighting that activities beyond traditional exercise modalities like running or cycling may also impact circulating eCB levels. Despite these promising findings, a significant gap identified in our review is the lack of studies examining the effects of behavioral interventions, such as acute exercise, on circulating eCB levels in children or adolescents. Given the widespread use of physical activity interventions in youth and their associated mental health benefits (Recchia et al., 2023), investigating eCBs as a biomarker could optimize these interventions.

## Conclusion

The eCB system appears to be integral to numerous psychological processes, including stress, fear, emotion regulation, and social processing, which are critical to the emergence and progression of various psychiatric disorders. Preliminary evidence, particularly from animal models and adult studies, has provided valuable insights into eCB signaling. Clinical trials have yielded mixed results, highlighting a gap between preclinical discoveries and practical implications. This discrepancy underscores the need for a deeper understanding of the eCB system's complex interactions with psychopathology and developmental processes. Additionally, emerging research shows significant developmental changes in the eCB system. These changes are crucial for understanding how eCB signaling influences psychiatric risk and can be targeted via interventions at different life stages. Future studies, particularly longitudinal studies, are needed to track circulating eCB concentrations and their impact on mental health across the life span. Studies should investigate how eCB concentrations fluctuate during sensitive developmental periods and explore nonpharmacological approaches that may modulate eCB concentrations and offer therapeutic benefits. Understanding the eCB system's role in psychiatric risk during development may provide new intervention strategies, improve treatment outcomes, and reduce the burden of psychiatric disorders.

**Key points**

- The endocannabinoid system has been implicated in many mental health disorders.
- The endocannabinoid system is in flux during critical developmental periods throughout childhood and adolescence, and perturbations to this system from stress during these periods could impact future psychopathology.
- Pharmacological therapies targeting the endocannabinoid system, such as FAAH and MAGL inhibitors, can potentially provide the psychologically relevant benefits of cannabis without the psychoactive or putative neurodevelopmental effects.
- The development of the endocannabinoid system and its effects on child and adolescent psychopathology is a research area ripe for potential clinical translation.

## Data Availability

Data sharing not applicable to this article as no datasets were generated or analyzed during the current study.
